# Neonicotinoid pesticides: evidence of developmental neurotoxicity from regulatory rodent studies

**DOI:** 10.3389/ftox.2024.1438890

**Published:** 2024-10-02

**Authors:** Jennifer Beth Sass, Nathan Donley, William Freese

**Affiliations:** ^1^ Natural Resources Defense Council, New York, NY, United States; ^2^ Center for Biological Diversity, Portland, OR, United States; ^3^ Center for Food Safety, Washington, DC, United States

**Keywords:** pesticide, neurotoxic, DNT, neonicotinoid, EPA-environmental protection agency, neurodevelopment, developmental, brain

## Abstract

Neonicotinoids are the most widely used class of insecticides in the United States (U.S.). and the world. Consistent with their high use and persistence, neonicotinoids are often found contaminating drinking water and food. They are also detected in human urine, breast milk, amniotic and cerebrospinal fluids, as well as the brains of treated rodents. Neonicotinoids were once thought to pose little neurotoxic risk to humans, but a growing body of research challenges that assumption. In this study we provide the first comprehensive assessment of unpublished rodent developmental neurotoxicity (DNT) studies on five neonicotinoids that were submitted to the U.S. Environmental Protection Agency (EPA) by neonicotinoid manufacturers. Groups of female rats were administered three different doses of a neonicotinoid during pregnancy and lactation, and their offspring subjected to various neurological tests and brain measurements. We identified nicotine-like effects such as reduced brain size, indicative of neuronal cell loss. Statistically significant shrinkage of brain tissue was observed in high-dose offspring for five neonicotinoids: acetamiprid, clothianidin, imidacloprid, thiacloprid, and thiamethoxam. Two brain regions reduced in the rodent studies–the corpus callosum and caudate-putamen–tend to be smaller in people diagnosed with attention-deficit hyperactivity disorder (ADHD), and in children of mothers who smoked during pregnancy, suggesting a possible link between perinatal neonicotinoid exposure and ADHD. A decreased auditory startle reflex was reported for acetamiprid at all doses and was statistically significant in the mid- and high-dose offspring, and for clothianidin in juvenile high-dose females. No mid- or low-dose brain morphometric data were submitted for acetamiprid, imidacloprid, or thiacloprid. Thiamethoxam mid- and low-dose brain morphometric data were provided to EPA upon request. Only partial mid-dose brain morphometry data were submitted for clothianidin, but no low-dose data. Yet despite this lack of data, EPA concluded that only the high-dose brain morphometric effects were treatment-related–setting the mid-dose as the study’s No Observed Adverse Effect Level (NOAEL) or failing to find a definitive NOAEL for acetamiprid, clothianidin, imidacloprid, thiacloprid and thiamethoxam. We found numerous deficiencies in EPA’s regulatory oversight and data analyses. EPA dismissed statistically significant adverse effects, accepted substandard DNT studies despite lack of valid positive control data, and allowed neonicotinoid registrants to unduly influence agency decision-making. We conclude that perinatal exposure to neonicotinoids and their metabolites induces adverse, nicotine-like neurotoxic effects in rodent bioassays, and that the exposure limits set by EPA for human exposure are either not protective or not supported by available neurotoxicity data. We propose regulatory changes to empower EPA to better protect public health from developmental neurotoxins like neonicotinoids.

## Introduction

Most major classes of insecticides act by disrupting the nervous system through pathways that are conserved across invertebrate and vertebrate species (U.S. EPA, 2024b). For instance, both the organophosphate (OP) and the carbamate classes of insecticides are designed to disrupt cholinergic nerve function (Soltaninejad and Shadnia, 2014). Similarly, a newer class of insecticides, the neonicotinoids (neonics), act as cholinergic receptor agonists by binding to nicotinic acetylcholine receptors (nAChRs), which results in the opening of calcium and other cation channels. By this mechanism the neonicotinoid pesticides exert their lethal effect on invertebrates (Tomizawa and Casida, 2003).

**FIGURE 1:**
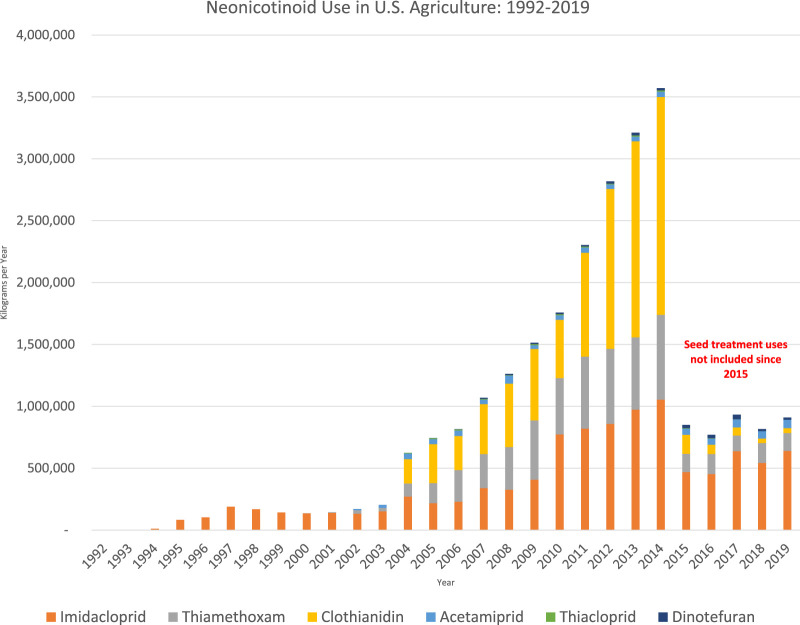
U.S. Geological survey data on neonicotinoid use (Wieben, 2021). Seed coating uses included up through 2014; Kynetec, the firm that supplies USGS with usage data, stopped reporting the amounts of neonicotinoids in seed coatings in 2015.

Neonics are now the most widely used insecticides in the US and globally with over three-quarters of neonicotinoids used as seed treatments, coated onto seeds of crops before dispersal (see Figure 1) (Douglas and Tooker, 2015; Douglas et al., 2024). Neonicotinoid seed coatings have dramatically expanded the amount of farmland treated with insecticides: at least 150 million acres in 2012 (Steeger, 2014), six times the amount of land treated with the top ten insecticides combined in 2001 (U.S. EPA, 2024b). Non-agricultural uses of neonics include lawns and gardens, parks and playgrounds, indoor bed bug treatment, and flea and tick treatments for pets.

With such widespread use, neonics routinely contaminate: waterways and tap water (Goulson, 2013; Klarich Wong et al., 2019; Millemann et al., 2020; Aggarwal, 2021); foods including fruits, vegetables and baby foods (Craddock et al., 2019; Zhang et al., 2019; USDA, 2022); and even human breast milk (Chen et al., 2020). Based on these food and water monitoring reports, it seems likely that a child growing up today may have been exposed to neonic pesticides during fetal development from *in utero* exposure, in infancy from contaminated breast milk and formula reconstituted with neonic-contaminated tap water, and into childhood from consuming contaminated drinking water and baby foods. Programs that conduct pesticide food and water monitoring, as well as biomonitoring, should continue and be expanded.

Given the potential for people to be regularly exposed to neonicotinoids, including during vulnerable periods of early life development, it is important to ensure that risk evaluations and regulatory approval of these neurotoxic insecticides meet (and hopefully exceed) the legal protections required by federal pesticide law.

The rodent Developmental Neurotoxicity Study (DNT) is one of several studies EPA uses to determine whether a pesticide poses a particular risk when exposures take place during early development of the brain and nervous system. This is because fetal and early infant life is when the mammalian brain and nervous system is being built. Neurotransmitters and their receptors help coordinate the process; they promote cell replication, initiate differentiation into different cell types, trigger then terminate formation of axons and synapses, regulate cell death and promote cell migration to specific brain regions to form the final architecture of the brain (England et al., 2017; Loser et al., 2021). If this complex and fragile developmental process is disrupted by xenobiotics, there is little opportunity for repair, and the damage can be permanent (Rice and Barone, 2000). The DNT study is known as a “guideline” study because it follows standardized Test Guidelines (U.S. EPA, 1998; OECD, 2007) to provide regulatory agencies with information needed to determine dose-response values and exposure limits.

Generally speaking, EPA sets maximum limits for acute (one-time) and chronic (lifetime) exposure by first deriving a no observed adverse effect level (NOAEL) from one or more guideline animal studies conducted with a pesticide. To set the human exposure limit, EPA divides the NOAEL by an uncertainty factor that is normally 100 (10 for interspecies extrapolation from a rodent study, and 10 for intraspecies differences across the human population) (U.S. EPA, 2002c). However, neither factor accounts for the greater susceptibility to pesticidal harm when exposure occurs *in utero* or in early life.

The Food Quality Protection Act (FQPA) of 1996 mandated that EPA consider available information concerning “the special susceptibility of infants and children,” including “neurological differences between infants and children and adults, and effects of *in utero* exposure to pesticide chemicals,” to ensure a “reasonable certainty” that “no harm” will result “from aggregate exposure” to a pesticide, including “all anticipated dietary and all other exposures for which there is reliable information” (FQPA, 1996).

The FQPA child protective factor is one way EPA can ensure a margin of protection–by reducing allowable exposure by a factor of 10 to account for the greater susceptibility of the young to developmental toxicants (EPA, 2002a). FQPA puts the burden of proof squarely on EPA to ensure that all uses of a pesticide meet the “reasonable certainty of no harm” standard for the general population and for every age group of children, including aggregate exposures from food, drinking water, and all household uses such as flea treatments for pets. The FQPA also mandates cumulative assessment of pesticides that share a common mechanism of toxicity. By law, EPA can modify or eliminate the FQPA 10X safety factor “only if, on the basis of reliable data, such margin will be safe for infants and children.” (FQPA, 1996). Unfortunately, EPA has too often reduced or removed this important child-protection factor from its pesticide assessments, including for the neonicotinoids (Naidenko, 2020).

## Evaluation of registrant developmental neurotoxicity studies

As part of implementing the FQPA, the EPA has required pesticide manufacturers (called “registrants”) to conduct a rodent test following a guideline specifically for DNT (U.S. EPA, 1998; OECD, 2007). The guideline method specifies that groups of female rats are fed differing doses of the test substance during pregnancy and lactation to assess potential effects on the neurological development through adulthood (postnatal day 60 or later) of offspring exposed *in utero* and in mother’s milk. The DNT guideline test includes neuropathology assessments, neurobehavioral endpoints, and body weight and other parameters common to other toxicity studies. There are four test methods for behavior: a functional observation battery (FOB); an open-field locomotor test to measure motor activity; an auditory startle test that measures the reflexive response to intense acoustic stimuli; and some tests for learning and memory such as a water maze test and a passive avoidance test. Developmental landmarks are recorded, including the ability to roll and reflexes for surface righting, time of eye opening and pupil constriction reflexes, and vaginal patency in females and preputial separation in males. Post-mortem observations include brain weight and brain histology to evaluate potential neuropathology. At postnatal day (PND) 11 and study termination, morphometric analysis to assess structural development of the brain is performed on various brain regions, such as structures within the neocortex, hippocampus and cerebellum, as well as subcortical regions like caudate-putamen and corpus callosum (U.S. EPA, 1998). The DNT study can be used to establish an acceptable exposure threshold for an acute (single) exposure, known as the acute reference dose, because “there is a presumption that effects during development may result from a single exposure” (U.S. EPA, 2002c).

Here we evaluate EPA’s DNT Data Evaluation Records (DERs), comprehensive evaluations prepared by EPA staff, for five unpublished DNT studies submitted to EPA by neonicotinoid manufacturers.

Although EPA produced a summary data report for dinotefuran, we did not include it in our analysis because it did not record any significant adverse effects. EPA noted that there were no adverse effects on litter number or offspring viability at the high dose, and that there were no deficiencies with the study; it was classified by EPA as “acceptable” (U.S. EPA, 2013).

Below and summarized in Table 1, we report evidence of brain tissue thinning in at least some of the offspring in the high dose treatment group of DNT studies with all five of the neonics we analyzed–acetamiprid, clothianidin, imidacloprid, thiacloprid, and thiamethoxam. The DNT guideline only requires that brain morphometric data be submitted to EPA for the mid and low dose groups if there is pathology in the high dose group. Unfortunately, other than for thiamethoxam, EPA received either no data or only partial data from each registrant for the mid and low dose groups. Even lacking brain morphometric data for mid and low doses, EPA presumed that the effect was only at the high dose. Other endpoints where EPA reported statistically significant adverse effects are discussed, and identified in the Table as “yes.” Where no statistically significant effects were identified, the space is left blank in the Table.

**TABLE 1:** Summary of significant adverse findings identified by EPA Data Evaluation Records (DERs) of Registrant-sponsored Developmental Neurotoxicity (DNT) studies for neonicotinoid pesticides.

		Acetamiprid	Clothianidin	Imidacloprid	Thiacloprid	Thiamethoxam
Brain tissue thinning	High dose	Yes	Yes	Yes	Yes	Yes
	Mid dose	No data	Partial data	No data	No data	Yes
	Low dose	No data	No data	No data	No data	Yes
Decreased auditory startle reflex	High dose	Yes	Yes			
	Mid dose	Yes	Yes			
	Low dose	Yes				
Decreased motor activity	High dose		Yes	Yes		
	Mid dose			Yes		
	Low dose					
Learning and behavior effects	High dose				Yes	
	Mid dose				Yes	
	Low dose					
Delayed sexual maturation	High dose				Yes	Yes
	Mid dose				Yes	Yes
	Low dose					Yes

### Acetamiprid

In a 2003 DNT study sponsored by Nippon Soda (Nemec, 2003), acetamiprid was administered via gavage to pregnant/lactating rats at doses of 0, 2.5, 10 or 45 mg/kg/day from gestation day 6 (GD6) through PND21. The study was first assessed in 2004, then revisited in 2007 and 2008 in response to the sponsor’s objections (U.S. EPA, 2008). EPA reviewers were unable to conclude whether or not acetamiprid affected learning or memory due to high variability in the test results, and effects on motor activity were uncertain due to problems with the control data: namely, that the normal developmental pattern for locomotion and motor activity was not seen in male control animals, and that the level of motor activity in control males seemed high. Brain morphometric data were only provided for the control and high dose animals. At PND72, the length of the ventral limb of the dentate hilus of the hippocampal formation was reduced by 15% in both male and female offspring, a statistically significant finding in the females (U.S. EPA, 2008).

The agency identified a dose-responsive decrease in the maximum auditory startle response in male offspring of all dosage groups at both timepoints (PND20/PND60): low-dose 15%/10%; mid-dose 27%/40%; and high-dose 42%/53% (U.S. EPA, 2008). The EPA’s statistical analysis identified the mid-dose as a significant effect level when data from male and female pups from PND20 and 60 were combined. The registrant contested EPA’s conclusions in a rebuttal report, arguing that the mid-dose was a no-effect level based on statistical analyses by two consulting groups (U.S. EPA, 2008). EPA statisticians rejected the consultants’ analyses due to inappropriate use of models and statistical errors (U.S. EPA, 2008). The European Food Safety Authority (EFSA et al., 2024) likewise rejected the pesticide industry’s statistical interpretation and set a no-effect level at the low dose of 2.5 mg/kg/day (EFSA, 2015). With the standard 100X uncertainty factor and the 10X FQPA child protective factor, the maximum acute exposure level regarded as safe for infants and children, known as the acute population, adjusted dose (aPAD), would be 2.5/1,000 = 0.0025 mg/kg/day.

In 2008, the EPA without explanation overruled its own statisticians’ conclusions and raised the offspring NOAEL to the mid-dose of 10 mg/kg/day in accordance with the registrant’s request (U.S. EPA, 2008). In 2017, EPA removed the FQPA child protective factor (reduced from 10X to 1X) in part based on the DNT study (U.S. EPA, 2017). These two changes together increased the aPAD by 40-fold to 0.1 mg/kg/day.

In Table 2, we show how these different aPADs result in radically different risk pictures (see Table 2 footnote). Based on EPA’s upperbound estimates of acute dietary exposure to acetamiprid, infants and children are exposed to 64%–87% of EPA’s official aPAD–where anything under 100% is considered acceptable (U.S. EPA, 2017). In contrast, that same exposure level exceeds a protective aPAD of 0.0025 mg/kg/day by a substantial 25–35-fold. Details on how EPA calculates dietary risk is in the 2017 acetamiprid draft human health risk assessment (U.S. EPA, 2017). In brief, dietary exposure is calculated as the combined exposure from both food residues and drinking water sources. Drinking water levels are predicted for both surface and groundwater sources using models. Food exposure is predicted using models populated with food consumption data from the US Department of Agriculture’s survey of “What We Eat in America.” Age-adjusted body weights and ingestion factors come from EPA’s Exposure Factors Handbook (U.S. EPA, 2024a).

**TABLE 2:** Summary of acute dietary exposure and risk estimates for acetamiprid.

Population subgroup	Acute Exposure (mg/kg/day) at the 95th Percentile^*^	% of Maximum “Safe” Exposure (aPAD) (mg/kg/day)	
		EPA aPAD = 0.1	Protective aPAD = 0.0025
All infants (<1 year)	0.069137	69%	2765%
Children 1–2 years	0.086734	87%	3469%
Children 3–5 years	0.064385	64%	2575%

^a^ For acute exposure levels, see EPA. (2017), Table 5.4.5.1, p. 29.

The more protective aPAD we propose for acetamiprid, 0.0025 mg/kg/day, is similar to what the European Food Safety Authority (EFSA) is now proposing. EFSA is recommending that the acceptable daily intake (ADI) of acetamiprid be lowered from 0.025 down to 0.005 mg/kg per day, to be more protective of potential developmental neurotoxicity risks (EFSA et al., 2024). EFSA’s 84-page report supporting the recommendation includes the results of a systematic review of public literature discussing evidence of acetamiprid and DNT effects from both *in vitro* and *in vivo* studies, including ones cited in this manuscript. We refer readers to that report for further details.

### Clothianidin

In a DNT study sponsored by Takeda Chemical Industries in 2000, female rats were fed clothianidin in the diet from GD0 to PND22 at doses of 0, 13, 43 and 142 mg/kg/day during gestation (Hoberman, 2000).

EPA flagged several serious study deficiencies that, to our knowledge, were never remedied. The study sponsor failed to provide EPA with the brain morphometric data for the low dose group. At the mid-dose, morphometric data were provided for females but not for males. For the mid dose females the brain morphometry data was provided to EPA only as a mean of both brain hemispheres, instead of separately (U.S. EPA, 2005). EPA noted all this in its list of study deficiencies and requested that any additional morphometric measurements should be submitted to EPA. Additional study deficiencies noted by EPA included no mention of any test results for pupillary function such as constriction and response to light.

Of the 17 brain measurements taken at PND12 and termination, among the high dose animals, statistically significant differences were reported for 6 measurements in females (2 increased, and 4 decreased) and 3 measurements in males (two increased, and 1 decreased). At termination, the 4 reported differences were all decreases (3 in females, 1 in males), suggesting that by about 3 months of age (PND83-87) the neurodevelopmental effects of clothianidin may include a thinning of brain tissues.

For the mid dose brain morphometric data for females, there were no statistically significant effects on brain measurements (U.S. EPA, 2005). It remains unknown whether the conclusion may have been different had the registrant submitted data for the individual hemispheres, and if the male data had also been submitted. The study remains classified as deficient for lacking this information.

Despite this, EPA set the offspring neurotoxicity NOAEL at the mid-dose based on high-dose effects, including decreased motor activity (number and duration of movements) in male offspring, decreased auditory startle response in female offspring and, at termination (PND83-87), a 5% thinning of the hippocampal gyrus in both sexes and a 6% reduction in caudate putamen thickness in females (U.S. EPA, 2005).

### Imidacloprid

In a 2001 DNT study conducted by Bayer (Sheets, 2001), imidacloprid was fed to three groups of pregnant/lactating Wistar rats from GD0 to PND21. Doses during gestation were 0, 8, 19 and 55 mg/kg/day. After weaning, offspring were given untreated feed and evaluated until 75 days of age.

EPA identified two major treatment-related neurodevelopmental effects (U.S. EPA, 2002b). First, the thickness of the caudate/putamen brain region was reduced by 5.4% in high-dose female pups at PND11 and by 2% at study termination (PND70), described by EPA as a “persistent change” in this structure. Second, motor activity was reduced in high-dose male and female pups at PND17, and in female pups at PND21 (U.S. EPA, 2002b). Though not statistically significant, EPA regarded the reduced motor activity as treatment-related and adverse due to its consistency in both sexes and magnitude (31%–38%). In a separate review of the same study, the California Department of Pesticide Regulation found a significant 27% reduction in the thickness of the corpus callosum in high-dose females at PND11 (Cal, 2006). The corpus callosum effects were not identified or reported by EPA.

Bayer did not comply with an EPA directive to supply caudate/putamen morphometric measurements for low- or mid-dose female animals (U.S. EPA 2002b), as required by both EPA and OECD Test Guidelines (U.S. EPA, 1998, OECD, 2007). Despite not having adequate data to assess harmful effects on the caudate/putamen, corpus callosum or other brain structures at the low- and mid-dose, in 2002 EPA set the offspring NOAEL at the mid-dose (U.S. EPA, 2002b).

### Thiacloprid

In a 2001 DNT study sponsored by Bayer Corporation (Hoberman, 2001), female rats were administered thiacloprid in the diet from GD0 to PND22 at 0, 4.4, 25.6 and 40.8 mg/kg/day during gestation. Brain weight and neuropathology were assessed at PND12 and PND68-79. A number of brain regions were adversely affected in male offspring at the high dose, including statistically significant 4% reductions in the corpus striatum, a region that encompasses the caudate-putamen, at both PND12 and termination; a 14% reduction in the corpus callosum at PND12; and a 5% reduction in the dentate gyrus at termination. EPA noted that “a definitive NOAEL was not established for these findings” given the lack of data for the mid- and low-doses (U.S. EPA, 2003a).

In tests of passive avoidance and behavior retention, females showed significantly poorer performance at the mid-dose and high-dose treatments compared with controls (U.S. EPA, 2003a). EPA identified “suggestive effects” on motor activity and auditory startle reflex in both the mid- and high-dose groups that were not statistically significant.

Sexual maturation was statistically significantly delayed by an average of a half to a full day in the mid and high dose male pups (as measured by preputial separation), and in the high dose female pups (as measured by vaginal patency) (U.S. EPA, 2003a). These are measurements of hormone-dependent developmental landmarks of sexual maturity that occur at the time of puberty in both rats and humans.

EPA’s documented concerns with the positive control data that was submitted with the study were substantial: “Most of the positive control studies are unacceptable for use with the current study. … None of the studies demonstrated the laboratory’s ability to detect major functional neurotoxic endpoints using the observational methods used in the current study.” (U.S. EPA, 2003a). EPA's list of study deficiencies was a page long, with the lack of acceptable positive control data listed last. Other study deficiencies identified by EPA included: inadequate description of the methods used to evaluate functional behavior; motor activity never habituated, with no explanation provided; the termination of the study with final brain pathology data was over an 11-day period, with no explanation for this wide range of ages at study termination; brain measurements were made bilaterally but only reported as the mean value of both hemispheres; although treatment-related alterations in brain morphology were reported for the high-dose, the brain morphometry at the mid and low dose levels were required but were not received.

The study was judged “unacceptable” due to numerous serious deficiencies, including failure to supply brain morphometry, for low- and mid-dose groups. Because of this, EPA could not set a definitive NOAEL for offspring and arbitrarily applied a 3X “database uncertainty factor” in calculating the effect concentration (U.S. EPA, 2003b).

### Thiamethoxam

In a 2003 DNT study sponsored by Syngenta Crop Protection Inc. (Brammer, 2003), thiamethoxam was administered in the diet to female rats from GD7 to PND22 at doses of 0, 4, 35 and 300 mg/kg/day during gestation. Brain morphometry was conducted on high-dose animals sacrificed at PND12 and at study termination on PND63. Upon request by EPA, Syngenta submitted mid- and low-dose brain morphometric data, which were analyzed in a separate DER in 2007 (U.S. EPA, 2007).

Thiamethoxam reduced brain weight significantly at termination in high-dose males and females as well as mid-dose females. Of the 14 brain regions/parameters that were analyzed in the male offspring at termination, 12 of the high-dose parameters were significantly reduced (by 5%–20%) compared with control animals (U.S. EPA, 2007). At the mid-dose, 9 of the parameters were reduced in size compared with controls, 6 of the regions were reduced by 2%–13%, and 3 were statistically significant reductions. Among low-dose male offspring at termination, 6 of 12 regions were reduced in size (by 5%–15%), and 2 were statistically significant (U.S. EPA, 2007).

The most consistently affected brain regions across sexes and doses were the dorsal cortex, the thalamus, and the corpus callosum–the latter’s thickness reduced by 20% and 16% in high-dose males at termination and females at PND12, respectively (U.S. EPA, 2007). Significant changes in the male thalamus at termination included reduced height (high-dose), reduced width (mid- and high-dose), and decreased overall width of the thalamus/cortex (all doses). The thalamus width of females was significantly reduced in all dosage groups at PND12. The dorsal cortex thickness of males at termination was significantly reduced by 11%–15% in all dosage groups in one set of level 3 specimens, and by 6%–11% in high-dose males for three other sets of specimens (levels 3, 4 and 5).

Age at sexual maturation in male offspring (measured as preputial separation) was delayed across all thiamethoxam treatment groups, by an average of a half-day at the low dose and an average of 1.5 days at the high dose (U.S. EPA, 2007). The delay was statistically significant in the low (*p* < 0.05) and high dose group (*p* < 0.01), compared with control animals. EPA notes in its report that the study did not monitor or report on other developmental landmarks such as tooth eruption and ear pinna unfolding.

Despite the treatment-related effects in offspring of all dosage groups and both sexes, including reduced brain weight in mid-dose females, EPA concluded that only effects at the high dose were treatment-related and set the study offspring NOAEL at the mid-dose, 35 mg/kg/day (U.S. EPA, 2020).

## Discussion

Our review of the EPA data reports for rodent DNT studies consistently found a significant reduction in brain tissue in high-dose offspring for five neonicotinoids: acetamiprid, clothianidin, imidacloprid, thiacloprid, and thiamethoxam.

Additionally, reported effects of acetamiprid include reduced auditory startle reflex at all doses, with statistical significance in the mid- and high-dose groups. The clothianidin DNT also reported reduced auditory startle reflex in high-dose juvenile females. Decreased motor activity was observed for clothianidin (high-dose males) and imidacloprid (high-dose in both sexes). The thiamethoxam DNT recorded delayed sexual maturation in male offspring across all doses that was statistically significant at the low and high dose. Thiacloprid was associated with poor behavior retention in mid- and high-dose females, and with delayed sexual maturation in the mid and high dose male pups, and in the high dose female pups (See Table 1).

Because the study sponsor failed to submit to EPA the required brain morphometric data for mid- or low-dose groups for acetamiprid, imidacloprid, or thiacloprid, a true NOAEL for the morphometric brain effects cannot be determined. Thiamethoxam’s mid- and low-dose data were supplied to the EPA upon request. For clothianidin only the female mid-dose data were given to EPA, but not male mid-dose or the low dose for either sex. Despite these data gaps, EPA designated the mid dose (for which in most cases it had no data) as the NOAEL for all five neonic pesticides. In addition to the obvious problems with determining a NOAEL without supportive data, in some cases this determination was contrary to the recommendations of the scientist that reviewed the data (acetamiprid) or was made despite a lengthy list of concerns regarding study deficiencies (thiacloprid).

The precise mechanisms of the effects we identified are unclear, and it is beyond the scope of our study to explore them in detail (the regulatory DNT studies are intended only to identify endpoints associated with developmental neurotoxicity and to quantify potential differences in life-stage susceptibility, not investigate mode of action.) However, some insights might be gleaned from the extensive body of research on nicotine, a well-established developmental neurotoxin (Slotkin, 2008; England et al., 2017; Castro et al., 2023), based on their extensive similarities.

### Nicotine-like effects of neonicotinoids on the cholinergic system in neurodevelopment

Neonicotinoids are similar in structure to nicotine, and like it are agonists of nicotinic acetylcholine receptors (nAChRs) (Kimura-Kuroda et al., 2012). Neonicotinoids penetrate the blood-brain barrier (Hirano et al., 2021; Katić et al., 2021) and access the fetal brain (Burke et al., 2018) in animal models. They are detected in human cerebrospinal fluid (Laubscher et al., 2022; Li et al., 2022), pass through the human placenta (Zhang et al., 2022), and are found in the breast milk of lactating women (Zhang et al., 2023). Fetal exposure to nicotine via maternal smoking has long been established (Luck et al., 1985).

The results of these DNT studies contribute to the growing evidence that neonicotinoids exert adverse, nicotine-like effects on the developing mammalian brain (Cal, 2006; Kimura-Kuroda et al., 2012). The reported dimensions of certain brain regions were nearly all smaller in adult offspring exposed perinatally to neonicotinoids, while overall brain weight declined in response to thiamethoxam. Reduced volume of the developing brain is a sensitive indicator of neuronal cell loss from exposure to developmental neurotoxicants (Kaufmann and Gröters, 2006). These findings are consistent with studies showing reduced neurogenesis and increased neuronal cell death in the hippocampus of neonatal mice exposed to either imidacloprid or acetamiprid (Nakayama et al., 2019), and decreased neurogenesis in mouse embryos following prenatal exposure to acetamiprid (Kagawa and Nagao, 2018).

Imaging studies have shown that fetal brain exposure to nicotine via maternal smoking during pregnancy also reduces human brain volume and the dimensions of certain brain regions (Anblagan et al., 2013; England et al., 2017), likewise via neuronal cell damage and death (Slotkin, 2008). And while maternal smoking involves perinatal exposure to many bioactive compounds in tobacco smoke that suppress overall fetal growth, animal models involving exposure to nicotine alone demonstrate nicotine-specific, cholinergic effects on fetal brain development at very early stages of development, even when subsequent birth weight is normal (England et al., 2017). Importantly, reduced brain dimensions in the rat DNT studies persisted in adult offspring (PND 63-87). Perinatal nicotine exposure likewise can cause changes in the trajectory of brain development that persist into maturity (Slotkin, 2008).

These similarities in the effects of neonicotinoids and nicotine on mammalian brain size beg the question of whether they may also trigger similar neurobehavioral outcomes.

As discussed above, the reduced brain dimensions in the DNT rat studies were accompanied by functional nervous system deficits: decreased auditory startle reflex, decreased motor activity, and impaired learning, suggesting a possible link between brain effects and neurobehavioral outcomes. Interestingly, auditory processing defects are also effects of *in utero* nicotine exposure (Dwyer et al., 2008).

The brain structures most consistently reduced across rodent DNT studies were the corpus callosum and the caudate-putamen. The corpus callosum is a bundle of nerve fibers that connects the right and left hemispheres and processes sensory, motor and high-level cognitive signals (Goldstein et al., 2024). The caudate-putamen is part of the dorsal striatum, which is primarily involved in control over conscious motor movements and executive functions (Young et al., 2024). The neonicotinoid-induced reduction of these structures in rodent studies suggests a possible link to attention-deficit hyperactivity disorder (ADHD) in humans, for several reasons. First, imaging studies seeking neuroanatomical correlates of ADHD have found that people with clinically diagnosed ADHD tend to have smaller corpus callosa (Hynd et al., 1991; Giedd et al., 1994; Baumgardner et al., 1996; Semrud-Clikeman and Bledsoe, 2011; Yu et al., 2023), and in some studies reduced volume of the caudate-putamen as well (Valera et al., 2007; Emond et al., 2009). While these studies did not investigate potential causal factors, others have found a decrease in corpus callosum thickness in children born to mothers who smoked during pregnancy—suggesting a potential link with nicotine—in some cases accompanied by lack of coordination during information and auditory process (Bublitz and Stroud, 2012). Two additional studies find the corpus callosum reduction only in female (Paus et al., 2008) or male (Björnholm et al., 2020) children of maternal smokers. Finally, others have identified smoking during pregnancy as a risk factor for ADHD in their children, irrespective of possible anatomical anomalies of the brain (Milberger, et al., 1996; Milberger et al., 1998; Dong et al., 2018; Huang et al., 2018). That prenatal exposure to tobacco smoke (in humans) and neonicotinoids (in rats) both induce shrinkage of structures whose smaller size appears to be characteristic of ADHD, and that people having a mother who smoked during pregnancy is independently associated with ADHD, at least suggests the possibility that prenatal exposure to neonicotinoids in humans may increase risk of this disorder as well. While this hypothesis is largely correlational, it finds support in the common effects exerted by neonicotinoids and nicotine on mammalian brain development discussed above.

Of course, one must also consider exposure, and the fact that neonicotinoids show considerably less affinity for mammalian nAChRs than nicotine (Tomizawa and Casida, 2003). However, two neonicotinoids break down to form nicotinoid metabolites (desnitro-imidacloprid and descyano-thiacloprid) that have equal or greater potency as agonists of nAChRs in mammals relative to nicotine (Tomizawa and Casida, 2003). Imidacloprid is degraded to its desnitro form in the environment, in treated plants, and in the mammalian liver (Cal, 2006; Loser et al., 2021). Desnitro-imidacloprid is found in human urine (Wang et al., 2020) and in drinking water (Klarich et al., 2017). A preliminary risk assessment of dietary exposure to desnitro-imidacloprid in food concluded that internal levels could be high enough to activate nAChRs, and would even be more likely to desensitize these same receptors–with desensitization occurring at around 17 nM, roughly 10-fold lower than activating levels (Loser et al., 2021). This resembles the capacity of nicotine to desensitize rat nAChRs at the low, non-activating concentration of 10 nM (Paradiso and Steinbach, 2003). Neonicotinoid desensitization of nAChRs could be as problematic as activation, disrupting normal neuronal function and neurodevelopment (Loser et al., 2021) with potential effects on the operation of neural networks involved in memory and learning processes (Ochoa et al., 1989).

Because these metabolites of imidacloprid and thiacloprid have nicotine-like potency, one might expect to see neurodevelopmental impacts of exposure to their parent chemicals at low exposure levels. While we have not exhaustively reviewed the literature, two relevant studies conducted at doses near or below acute regulatory thresholds for human exposure stand out. Babeľová et al. (2017) orally exposed female mice to 0.03 mg/kg/day thiacloprid on days 1–3 of pregnancy, and found the isolated day 4 blastocytes exhibited significantly decreased cell numbers versus controls, cell loss that could ramify into neuronal cell deficits in the brain of developing fetuses. Saito et al. (2023) orally administered imidacloprid at 0.01 mg/kg/day or nicotine (0.1 mg/kg/day) to maternal mice from embryonic day 11 to 4 weeks after birth, and found that both imidacloprid and nicotine impaired certain aspects of learning and memory in male pups subjected to a water maze test.

### Developmental neurotoxicity studies provide critical information, but must be conducted and overseen competently

Industry and EPA scientists who support *in vitro* approaches to assess DNT (discussed below) have argued that brain morphometry is unreliable because it is prone to “technical artifact” (Jackson et al., 2024). Yet when properly performed, morphometric analysis of brains can supply valuable data for regulators and is associated with less variability than body weight (Crofton, et al., 2001), a commonly used endpoint. The full suite of DNT test methods have been extensively validated; can provide reliable, relevant and reproducible data; and represent the best available science for assessing DNT potential in humans (Makris et al., 2009). However, improvements are needed. An analysis of 69 pesticide DNT study results submitted to EPA found that among the neurobehavioral tests, cognitive function and the FOB were used the least to determine a LOAEL, suggesting that within the guideline test they are not sufficiently sensitive endpoints (Vorhees and Makris, 2015). Locomotor activity and auditory startle were used most frequently for setting a LOAEL. Vorhees and Williams (2024) recommended updates to the DNT with additional requirements for more sensitive tests of learning and memory, while also noting that additional guidance may be helpful to improve the rigor of testing and reporting of results.

Deficiencies in DNT study data that do arise are often attributable to poor performance. An EPA review of positive control studies (studies undertaken with positive control chemicals known to disrupt neurological development) from labs that perform DNT studies found very troubling deficiencies; for instance, only three of the 16 demonstrated proficiency in testing for all DNT endpoints (Crofton et al., 2004). For 4 of the 5 DNT studies reviewed here (excepting clothianidin), adequate positive control data had either not been received or fully evaluated by EPA at the time the DERs were written, potentially compromising the integrity and reliability of the test results. Indeed, for thiacloprid EPA noted that: “None of the [positive control] studies demonstrated the laboratory’s ability to detect major functional neurotoxic endpoints using the observational methods used in the current study.” (U.S. EPA, 2003a).

Poor scientific practices can also be perpetuated by deficient regulatory oversight. For instance, EPA flagged the failure to submit brain morphometry for mid- and/or low-dose animals as a study deficiency, but then went ahead and set a LOAEL at the high-dose and NOAEL at the mid-dose for acetamiprid, clothianidin, and imidacloprid with the presumption–in the absence of complete data–that there would be no adverse effects on the brain at the mid- and low-doses. We believe that such determinations should be based on data, not speculation. Other unremedied deficiencies identified by EPA included inadequate assessment of motor activity, learning and memory (acetamiprid), no reporting of criteria for scoring errors in the water maze tests (clothianidin and thiacloprid) and failure to report how functional observation assessments are conducted (clothianidin and thiacloprid). EPA thus accepts studies that it deems deficient and that may well miss important adverse neurological effects, and registrants face no consequences for failing to supply missing or inadequate data.

It is our opinion that the quality of rodent DNT and other regulatory toxicology studies would improve considerably if EPA were to reject seriously deficient studies, enforce requests for additional data, and cancel or refrain from approving or re-approving pesticides when reliable data are lacking.

### Developmental neurotoxicity studies moving forward

There is considerable momentum at EPA’s Office of Pesticide Programs to replace DNT rodent studies with new approach methodologies (NAMs) involving *in vitro* cell-based assays and *in silico* computational models (Crofton et al., 2014). The rationales most often cited are the time and expense of animal testing, and the laudable goal of reducing animal suffering (Crofton et al., 2014; Zaveri et al., 2019). However thus far, there is no adequate alternative to *in vivo* DNT studies (Vandenberg, and Zoeller, 2019). The OECD recently reviewed the DNT *in vitro* battery of tests (called the DNT IVB), warning that, “Several gaps in coverage of neurodevelopment processes and cell types have been acknowledged, including assays for neuroectodermal formation, peripheral nervous system specific processes, astrocyte differentiation and maturation, the blood-brain and placental barriers, microglia regulation of neuronal growth and connectivity, neuronal subtype specification, and axon myelination…. Also, the current DNT IVB does not fully account for sex or human genetic diversity that may influence susceptibility to chemical-induced developmental neurotoxicity (i.e., gene × environment interaction). These factors may result in lower sensitivity and specificity.” (OECD, 2023).

The European Partnership for the Assessment of Risks from Chemicals (PARC), which includes authors from 22 government agencies and academic institutions, published an article in Frontiers in Toxicology in April 2024 concluding that the current DNT NAMs have too many gaps to be used in risk assessment at this time (Tal et al., 2024). PARC particularly identified functional gaps, including tests of cognitive and neurobehavioral outcomes, cell processes within whole organisms, and learning and memory. The PARC report notes that these gaps will remain even with the future-planned DNT NAMs tests, unless additional whole animal tests are included using zebrafish.

Instead of investing in updating the rodent DNT tests to improve the quality, rigor, and sensitivity to detect complex neurodevelopmental effects such as learning, memory and behavior, EPA has placed its confidence in the DNT NAMs tests. EPA is so confident in NAMs that it is relying on a lack of bioactivity in NAMs tests as evidence of lack of DNT, leading to less-protective risk estimates for several organophosphate pesticides (U.S. EPA, 2023; U.S. EPA, 2024c). This misuse of NAMs is strongly opposed by health scientists and regulators alike (Children’s Health Protection Advisory Committee, 2021; Birnbaum, et al., 2024; Khan, 2024; Lam et al., 2024; Newell-Price, 2024).

### Regulatory recommendations

EPA should make DNT studies a core requirement for registration of every pesticide, as its own scientists recommended in 1999 (U.S. EPA, 1999). This would reverse a disturbing trend of DNT study waivers that EPA has granted and even celebrated in recent years (Craig et al., 2019; Lerner, 2021).

Given the clear evidence of neonicotinoids’ mammalian neurotoxicity, EPA should reduce the acute and chronic reference doses (exposure limits) for each of them by a factor of at least 10 to account for the special sensitivity of the developing nervous system, as mandated by the FQPA.

Because neonicotinoids and their metabolites share a common mechanism of toxicity with nicotine, EPA should conduct a cumulative assessment of these insecticides, as mandated by another provision of the FQPA. This could be accomplished by assigning each neonicotinoid and major metabolite a relative potency factor that accounts for the greater toxicity of certain metabolites.

The FQPA authorizes EPA to eliminate a 10X child protective factor only if it has reliable information to find reasonable certainty of no harm to children without that protection. Given the gaps in coverage and the lack of validation with DNT NAMs, the risks to human and environmental health, and scientific uncertainties are far too great for EPA to rely on negative results (no bioactivity results) from NAMs tests. Instead, EPA could follow a recommendation of its Children’s Health Protection Advisory Committee, and employ NAMs results only to indicate or upgrade concern for a hazard, but not to conclude absence of hazard or to reduce the margin of protection afforded by the FQPA 10X child protective factor (CHPAC, 2021).

## Conclusion

The rodent studies reviewed here provide valuable insights into the developmental neurotoxicity of five neonicotinoids, revealing similarities to the effects of nicotine, which is known to disrupt mammalian neurological development. Early-life exposure to each neonicotinoid reduced the dimensions of various brain regions, signifying neuronal cell death and reduced neurogenesis. Shrinkage of the brain regions most consistently affected across studies–the corpus callosum and caudate-putamen–suggests a possible role in the genesis of attention-deficit hyperactivity disorder (ADHD). The studies also demonstrated reduced auditory startle response and suggested adverse effects on learning and memory.

Further research is needed into the developmental neurotoxicity of neonicotinoids, and in particular metabolites equipotent to nicotine, especially given the ubiquitous use of and exposure to these compounds and the potential for life-long impairment. The conduct and oversight of regulatory DNT studies on neonicotinoids and other pesticides must be improved so they can provide higher-quality data. Well-conducted rodent studies of sufficient statistical power and strict adherence to required animal welfare protections remain critical for assessing xenobiotic disruption of complex neurodevelopmental processes. While new approach methodologies (NAMs) may contribute valuable insights into the cellular and molecular mechanisms of such adverse effects, they are not currently capable of replacing *in vivo* assessments.

## Summary

Neonicotinoid insecticides are widely used, environmentally persistent, and are detected in drinking water, foods, human urine, breast milk, amniotic and cerebrospinal fluids, and the brains of treated rodents. Here we provide the first comprehensive assessment of unpublished rodent developmental neurotoxicity (DNT) studies on five neonicotinoids sponsored by neonicotinoid manufacturers. Statistically significant shrinkage of brain tissue was observed in high-dose offspring for five neonicotinoids: acetamiprid, clothianidin, imidacloprid, thiacloprid, and thiamethoxam. A decreased auditory startle reflex was reported for acetamiprid at all doses and was statistically significant in the mid- and high-dose offspring, and for clothianidin in juvenile high-dose females.

## Data Availability

The original contributions presented in the study are included in the article/supplementary material, further inquiries can be directed to the corresponding author.
